# Geographical distribution of *Burkholderia pseudomallei* in soil in Myanmar

**DOI:** 10.1371/journal.pntd.0009372

**Published:** 2021-05-24

**Authors:** Myo Maung Maung Swe, Mo Mo Win, Joshua Cohen, Aung Pyae Phyo, Htet Naing Lin, Kyaw Soe, Premjit Amorncha, Thin Thin Wah, Kyi Kyi Nyein Win, Clare Ling, Daniel M. Parker, David A. B. Dance, Elizabeth A. Ashley, Frank Smithuis

**Affiliations:** 1 Myanmar Oxford Clinical Research Unit, Yangon, Myanmar; 2 Centre for Tropical Medicine and Global Health, University of Oxford, Oxford, United Kingdom; 3 Department of Medical Research, Ministry of Health and Sports, Yangon, Myanmar; 4 Mahidol-Oxford Tropical Medicine Research Unit, Faculty of Tropical Medicine, Mahidol University Bangkok, Thailand; 5 Shoklo Malaria Research Unit (SMRU), Mahidol-Oxford Tropical Medicine Research Unit, Faculty of Tropical Medicine, Mahidol University, Mae Sot, Thailand; 6 Department of Population Health and Disease Prevention Program in Public Health, University of California, Irvine, CA, United States of America; 7 Lao-Oxford-Mahosot Hospital-Wellcome Trust Research Unit, Vientiane, Lao People’s Democratic Republic; 8 Faculty of Infectious and Tropical Diseases, London School of Hygiene and Tropical Medicine, London, United Kingdom; University of Texas Medical Branch, UNITED STATES

## Abstract

**Background:**

*Burkholderia pseudomallei* is a Gram-negative bacterium found in soil and water in many tropical countries. It causes melioidosis, a potentially fatal infection first described in 1911 in Myanmar. Melioidosis is a common cause of sepsis and death in South and South-east Asia, but it is rarely diagnosed in Myanmar. We conducted a nationwide soil study to identify areas where *B*. *pseudomallei* is present.

**Methodology/Principal findings:**

We collected soil samples from 387 locations in all 15 states and regions of Myanmar between September 2017 and June 2019. At each site, three samples were taken at each of three different depths (30, 60 and 90 cm) and were cultured for *B*. *pseudomallei* separately, along with a pooled sample from each site (i.e. 10 cultures per site). We used a negative binomial regression model to assess associations between isolation of *B*. *pseudomallei* and environmental factors (season, soil depth, soil type, land use and climate zones). *B*. *pseudomallei* was isolated in 7 of 15 states and regions. Of the 387 sites, 31 (8%) had one or more positive samples and of the 3,870 samples cultured, 103 (2.7%) tested positive for *B*. *pseudomallei*. *B*. *pseudomallei* was isolated more frequently during the monsoon season [RR-2.28 (95% CI: 0.70–7.38)] and less in the hot dry season [RR-0.70 (95% CI: 0.19–2.56)] compared to the cool dry season, and in the tropical monsoon climate zone [RR-2.26; 95% CI (0.21–6.21)] compared to the tropical dry winter climate zone. However, these associations were not statistically significant. *B*. *pseudomallei* was detected at all three depths and from various soil types (clay, silt and sand). Isolation was higher in agricultural land (2.2%), pasture land (8.5%) and disused land (5.8%) than in residential land (0.4%), but these differences were also not significant.

**Conclusion/Significance:**

This study confirms a widespread distribution of *B*. *pseudomallei* in Myanmar. Clinical studies should follow to obtain a better picture of the burden of melioidosis in Myanmar.

## Author summary

Melioidosis is caused by a soil dwelling bacterium called *Burkholderia pseudomallei*. It is a potentially fatal disease even if appropriate antibiotics are given. The disease was first described in Rangoon (now Yangon) in 1911 but it has rarely been reported in Myanmar over the past century even though it is now known to be a common cause of community-acquired infection in neighbouring countries in South and Southeast Asia. The true melioidosis burden in Myanmar is obscured by a lack of awareness among clinicians and limited diagnostic facilities to identify the causative organism. To explore this issue, we conducted a nationwide study to assess the presence of *B*. *pseudomallei* in soil samples collected from all fifteen states and regions of Myanmar. Our study revealed a widespread distribution of the organism in the Myanmar environment. This finding contributes to the understanding of the global distribution of *B*. *pseudomallei* and should alert clinicians throughout Myanmar to consider melioidosis as a potential cause of fever, sepsis, pneumonia and abscesses.

## Introduction

Melioidosis is a serious tropical infection caused by the Gram-negative bacterium *Burkholderia pseudomallei*. It is endemic in tropical areas of South and South-east Asia and Northern Australia and is increasingly being detected in many other tropical regions.[1] It often presents with life threatening sepsis, pneumonia or abscesses in the internal organs, [2,3] but the presentation can also be uncharacteristic making it very difficult to differentiate from other diseases clinically, hence its nickname “the remarkable imitator”.[4–10] In addition, laboratory diagnosis is not straightforward and the organism is often dismissed as a contaminant or misidentified.[11,12]

*B*. *pseudomallei* is intrinsically resistant to a wide range of antibiotics (penicillin, ampicillin, first- and second-generation cephalosporins, aminoglycosides, macrolides and polymyxins) and routine treatment protocols for sepsis and pneumonia frequently do not cover *B*. *pseudomallei*.[3] Treatment usually comprises at least 10 days of parenteral antibiotics (ceftazidime or a carbapenem) followed by oral ‘eradication’ antimicrobials for 3 to 5 months.[13] Even with effective antimicrobial therapy, case fatality rates still reach up to 50%.[14] In 2016, it was estimated that 165,000 (95% credible interval 68,000–412,000) cases of melioidosis occurred annually with 89,000 (36,000–227,000) deaths worldwide [1], representing a higher disease burden than that associated with many infections classified as Neglected Tropical Diseases.[15]

*B*. *pseudomallei* can be found in soil and surface water of endemic regions [16–19] and infection is acquired by direct inoculation with contaminated soils, ingestion or aspiration of contaminated water or inhalation of dusts or aerosols. It occurs commonly in farmers or other people who are regularly exposed to soil and surface water.[20] People with underlying health conditions such as diabetes mellitus or chronic renal disease and patients on treatment with immunosuppressive drugs are at particular risk of contracting the disease.[20,21]

Melioidosis was first recognised in Rangoon (now Yangon), Myanmar in 1911.[22] Since then it has become recognised as an important public health problem in some countries, mainly in north Australia [21] and South East Asia.[23,24] However, a lack of awareness amongst clinicians and laboratory staff, and the poor availability of diagnostic facilities means that there is significant under-recognition.[1]

In Myanmar only 298 melioidosis cases have been reported since its first discovery in 1911 and most of these were identified before 1949.[25] However, it is unlikely that the bacterium has disappeared from the environment and the incidence is probably significantly underestimated. Limmathurotsakul *et al*. suggested that the burden of melioidosis in Myanmar was more than 6,000 cases and 3,000 deaths per year.[1]

Recently two small studies confirmed the presence of *B*. *pseudomallei* in the soil in three regions (Yangon, Ayeyarwaddy and Bago) [26,27] but the national distribution in Myanmar remains unknown. We therefore carried out a nationwide study to identify the geographical distribution of *B*. *pseudomallei* in soil in Myanmar. This information can be used as a basis for further investigation into melioidosis as a public health problem in Myanmar.

## Materials and methods

### Ethics statement

The study was approved by Ethics Review Committee, Department of Medical Research, Ministry of Health and Sports, Myanmar (Approval number: Ethics/DMR/2017/087).

### Study location

**Fig 1:**
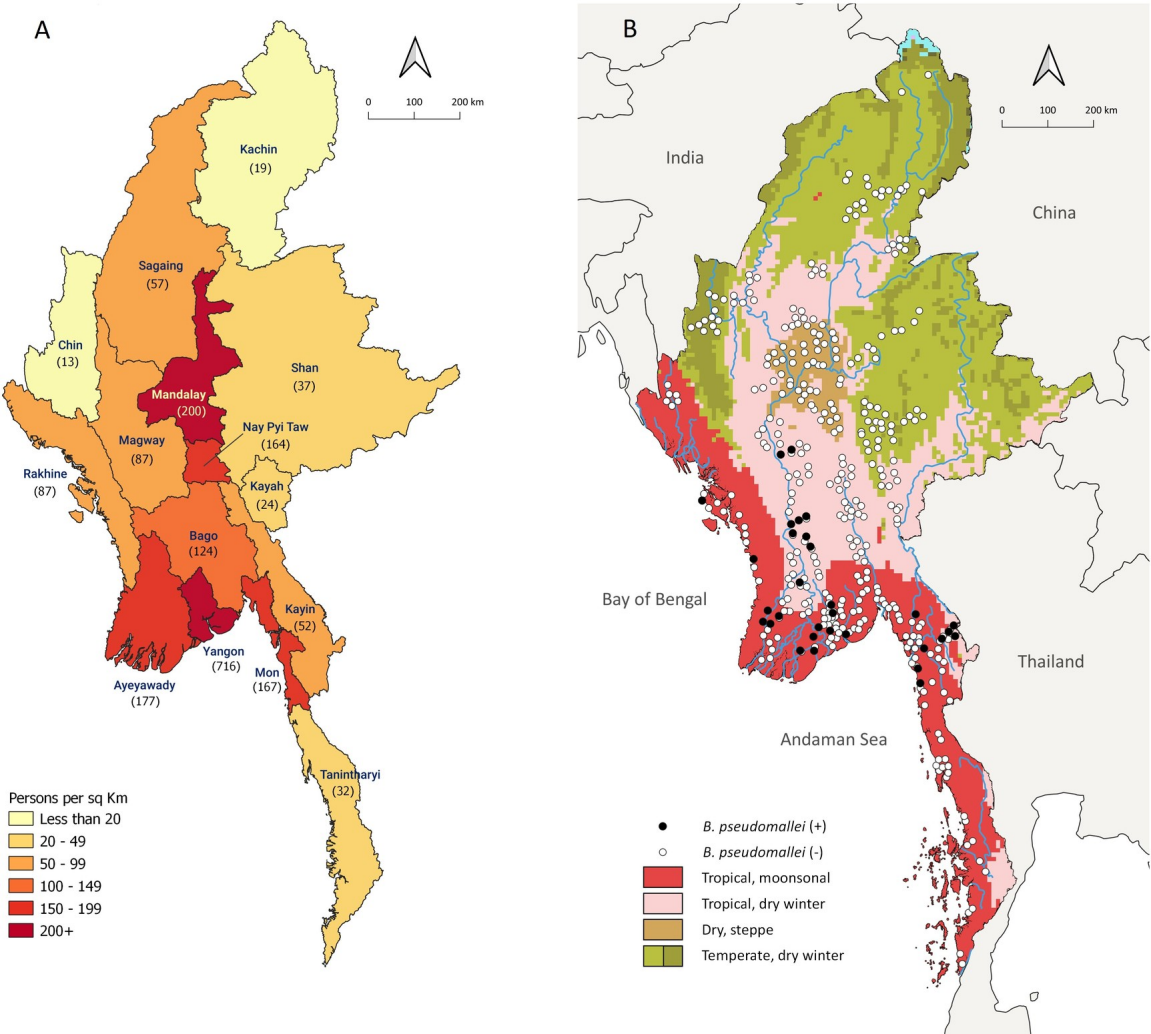
Distribution of *B*. *pseudomallei* positive sites across the country. A. Map of the states and regions of Myanmar with corresponding population density (person per square km). B. Map of Myanmar climate zones based on the Koppen Geiger climate classification (Source: http://koeppen-geiger.vu-wien.ac.at/present.htm). Black dots represent *B*. *pseudomallei*-positive sites (at least one sample positive for *B*. *pseudomallei*) and white dots represent *B*. *pseudomallei*-negative sites).

Myanmar is a lower middle income country situated in Southeast Asia with a population of approximately 54 million, of whom 70% live in rural areas.[28] The country has a diverse landscape with central lowland areas surrounded by mountains in the north, east and west, and coastline along the south. The climate is largely tropical, with a temperate climate in some areas in the North and a dry steppe climate in a small area in the centre of the country (Fig 1). The monsoon season is from mid-May to October, followed by a cool, dry season (November to February) and a hot, dry season (March to mid-May) [29].

### Sample site selection

We collected soil samples from all 15 states and regions of Myanmar; Kachin, Kayar, Kayin, Mon, Rakhine, Shan, Chin, Sagaing, Mandalay, Magway, Bago, Ayeyawady, Yangon, Tanintharyi and Naypyitaw. Eight hundred locations were generated by random sampling using a Geographic Information System (QGIS version 3.4.9 https://qgis.org/). These points were subsequently evaluated and 413 sites were excluded based on geographic characteristics (uninhabited forest, mountains or lakes), safety (armed conflict areas), and extreme remoteness (far away from accessible roads). We collected soil samples from the remaining 387 sites. At these sites, we selected locations purposively based on factors believed to be associated with the presence of *B*. *pseudomallei* and the presence of people, such as moist soil, animal grazing land and disused land within the proximity of a village. We classified the type of current land use into four categories; agricultural land used for rice and other plantation, pasture land (livestock grazing area), residential area and disused land. The soil type was classified by physical texture as sand, silt or clay. We selected more sites in the states and regions where population density is high (Ayeyawady, Bago, and Yangon), and in the states and regions with a larger geographic area (Sagaing and Shan). Overall, we collected soil samples from 161 of the 330 townships of Myanmar.

### Sampling method

The consensus guidelines for environmental sampling of *B*. *pseudomallei* recommends collecting 100 samples per site.[30] However, as we wanted to explore a very large geographical area, we chose to take fewer samples per site (10) in favour of a greater number of sites. At each selected site, a fixed interval sampling grid method was used to make nine holes (each hole five meters from its neighbours) at three different depths; three at 30cm, three at 60cm and three at 90cm, since the proportion of *B*. *pseudomallei*-positive samples at these different depths has varied in other studies. We used a mechanical auger to dig the holes at each selected site. The auger and shovel were cleaned with domestic water and 70% alcohol in between sites to avoid cross-contamination. Two samples (10 grams each) were collected from each hole. One sample was kept for culture while the second samples from each hole were pooled (weight 90 grams) and mixed on site before being sent for culture. We pooled samples in order to determine the sensitivity of pooled samples in comparison to individual samples as an attempt to simplify sampling procedures for detecting *B*. *pseudomallei* in soil. The 10 samples were kept in separate sealed plastic bags and stored at ambient temperature and away from direct sunlight. The samples were transported in a cool box and cultured within 48 hours at the Department of Medical Research in Yangon. Geographic coordinates (latitude and longitude), soil type (clay, silt, sand), and land use status were recorded at each sampling site.

### Isolation of B. pseudomallei

Ten grams of individual soil samples were mixed with 10 ml of threonine-basal salt solution plus colistin 50mg/L (TBSS-C50) in a universal tube.[30] The pooled 90 gram soil samples were mixed with 90 ml of TBSS-C50 solution. The tubes were vortexed for 30 seconds before incubating at 40°C for 48 hours. After that, 10 μl of the supernatant liquid was sub-cultured onto Ashdown agar and streaked to achieve single colonies. The plates were incubated at 40°C in air and checked daily for bacterial growth for the next 4 days. Colonies suspected of being *B*. *pseudomallei* were selected based on their typical appearance (purple colour, metallic sheen and rough/wrinkled surface). All such colonies underwent an oxidase test and a three disc (co-amoxiclav/colistin/gentamicin) screening test.[31] Colonies that had a positive oxidase test and a compatible antibiogram (resistance to gentamicin and colistin with susceptibility to co-amoxiclav) were confirmed as *B*. *pseudomallei* by a specific latex agglutination test and API 20NE.[32,33] Isolates with a compatible colonial appearance and antibiogram that were negative for latex agglutination and positive for arabinose assimilation in the Analytical Profile Index (API) 20NE were identified as *Burkholderia thailandensis*. The identity of a subset of seventeen isolates was confirmed by polymerase chain reaction (PCR) targeting the Type III Secretion System of *B*. *pseudomallei* using the method adapted and validated at Lao-Oxford-Mahosot Hospital-Wellcome Trust Research Unit from a previously published assay [34,35] and seven isolates were also confirmed by Whole Genome Sequencing (WGS).[36]

### Statistical analysis

We categorised the 15 states and regions into 3 climate zones based on the Koppen Geiger climate classification of Myanmar; the tropical monsoon zone along the coastal regions, the tropical dry winter/dry steppe zone in the central part of the country and the temperate dry winter zone in the north (Fig 1).[37] To investigate the association between environmental factors and isolation of *B*. *pseudomallei*, we used negative binomial regression to analyse the data. The outcome was the total number of positive *B*. *pseudomallei* samples across all depths at each site without clustering at hole level. We fitted univariable negative binomial regression model for the variables; climate zones (tropical monsoon, tropical dry winter/dry steppe and temperate dry winter), seasons (hot dry, monsoon and cool dry), soil type (clay, silt and sand), current land use (residential, agriculture, pasture land and disused land that did not vary at site level. To explore association between soil depth (three different depths at each site) and *B*. *pseudomallei* positivity, we analysed the outcome as the number of positive samples at site-depth level and fitted the site as random effect in the model to account for clustering of depths within each site. We did not perform multivariable regression because there were too few sites with positive samples (n = 31) to reliably estimate adjusted associations. We also fitted the model for a subset analyses between soil depth and season.

## Results

We collected 3870 soil samples from 387 sites in 161 townships across all 15 states and regions of Myanmar between September 2017 and June 2019. *B*. *pseudomallei* was isolated from 103 (3%) samples at 31 (8%) sites in 21 townships in 7 of the 15 states and regions (Tables 1 and S1). Ayeyawady, Kayin, Bago and Rakhine states and regions had the highest site positivity rates (defined as the percentage of sites with *B*. *pseudomallei* isolated from at least one of the 10 samples) at 9 of 36 (25%), 5 of 24 (22%), 6 of 37 (16%) and 2 of 13 (15%) sites respectively. The number of positive samples from each positive site ranged from a minimum of 1 to a maximum of 9 and the median (interquartile ranges) was 3 (1–4). Rakhine had the highest sample positivity rate with 15 of 130 (12%) samples, followed by Ayeyawady at 34 of 360 (9%), Bago at 18 of 370 (5%) and Kayin at 11 of 240 (5%). Although most *B*. *pseudomallei*-positive sites were located in the lower half of Myanmar, we did not detect *B*. *pseudomallei* in Tanintharyi province, located in the southernmost part of the country (Fig 1).

**Table 1:** Summary of population and land area together with number of sites and samples collected in each state and region.

States and Regions	Population	Land area (Km^2^)	Population density per 1000 Km^2^	Site positivity	Sample positivity	Season samples collected (year)
Yangon	7,360,703	10,277	716231	4/36 (11%)	13/360 (4%)	Rainy (2017)
Kayin	1,574,079	30,383	51808	5/24 (21%)	11/240 (5%)	Cool/dry (2017)
Mon	2,054,393	12,297	167065	3/26 (12%)	7/260 (3%)	Cool/dry (2018)
Kachin	1,689,441	89,042	18974	0/28	0/280	Cool/dry (2018)
Sagaing	5,325,347	93,702	56833	0/42	0/420	Hot/dry (2018)
Chin	478,801	36,019	13293	0/18	0/180	Hot/dry (2018)
Ayeyawady	6,184,829	35,032	176548	9/36 (25%)	34 /360 (9%)	Rainy (2018)
Bago	4,867,373	39,404	123525	6/37 (16%)	18/370 (5%)	Rainy (2018)
Mandalay	6,165,723	30,888	199615	0/24	0/240	Cool/dry (2018)
Magway	3,917,055	44,821	87393	2/29 (7%)	5/290 (2%)	Cool/dry (2018)
Tanintharyi	1,408,401	43,345	32493	0/20	0/200	Cool/dry (2019)
Rakhine	3,188,807	36,778	86704	2/13 (15%)	15/130 (12%)	Hot/dry (2019)
Kayar	286,627	11,732	24431	0/9	0/90	Hot/dry (2019)
Shan	5,824,432	155,801	37384	0/36	0/360	Hot/dry (2019)
Naypyitaw	1,160,242	7,057	164410	0/9	0/90	Rainy (2019)
**Grand total**	51,486,253	676,578	76098	31/387 (8%)	103/3870 (3%)	

**Fig 2:**
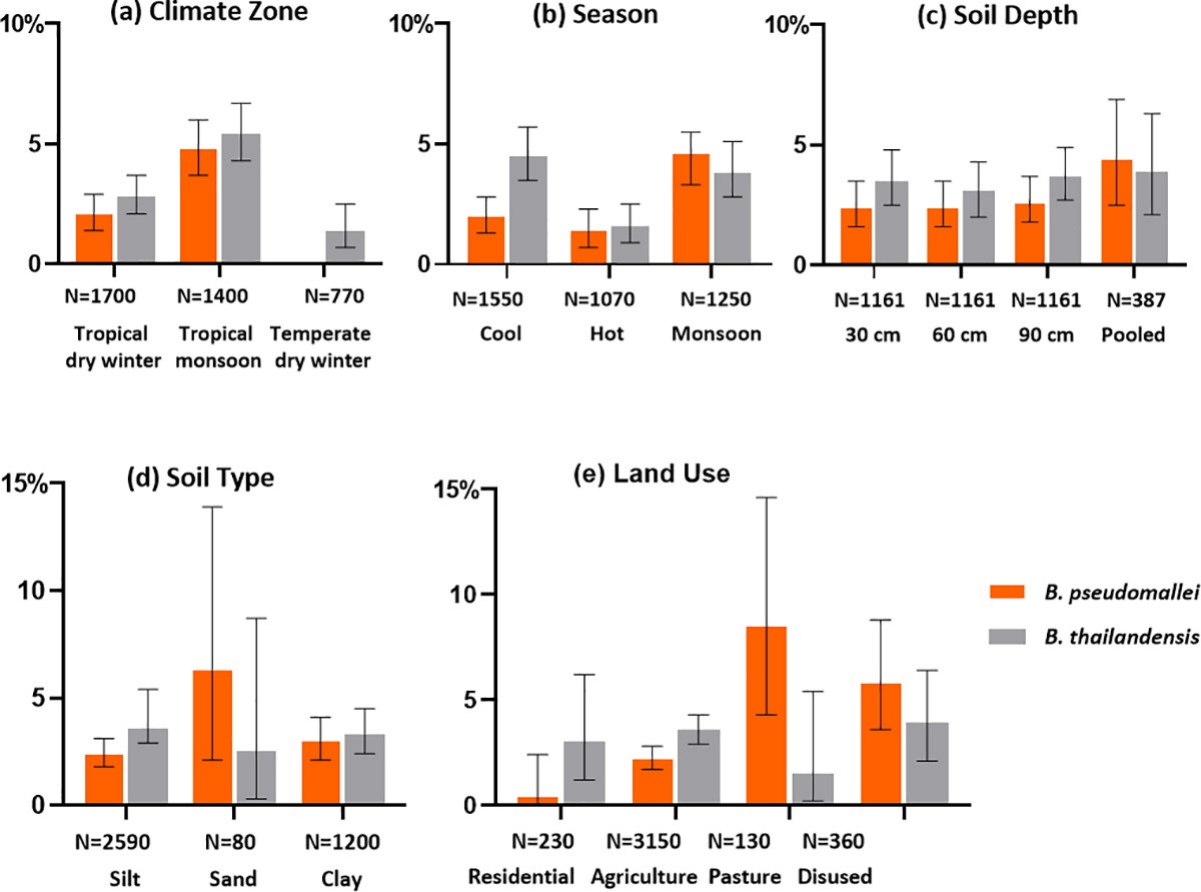
**Bar-plot showing percentage of sample positive for *B*. *pseudomallei* and *B*. *thailandensis* in different categories of (a) Climate zone (b) Season (c) Soil depth (d) Soil type and (e) Land use.** The numbers below each category represent the total number of samples collected in the corresponding category.

Details of soil sample culture results related to the climate zone, season of sample collection, soil type, current land use and soil depth are presented in Fig 2 and S2 Table. The results of univariable negative binomial regression analysis are presented in Table 2. We also fitted the model for subset analyses between soil depth and season, and the results are presented in Table 3. In terms of climate zones, 1700 (43.9%) and 1400 (36.2%) of samples were collected from tropical dry winter zone, largely along the main rivers in the central region, and the tropical monsoon zone in the coastal region, while 770 (19.9%) of samples were collected from the temperate dry winter zone in the northern regions (S2 Table).

**Table 2:** Association between *B*. *pseudomallei* isolation in the soil samples and season, soil depth, soil types, current land use and climate zones.

	Unadjusted rate ratio (95% Confidence Interval)	p-value
**Climate zones**		
Tropical dry winter/dry steppe	*Reference*	
Tropical monsoon	2.26 (0.21–6.21)	0.11
Temperate dry winter^1^	NA	
**Season**		
Cool, dry	*Reference*	
Hot, dry	0.70 (0.19–2.56)	0.59
Monsoon	2.28 (0.70–7.38)	0.17
**Soil depth**		
30 cm	*Reference*	
60 cm	1.03 (0.49–2.17)	0.95
90 cm	1.12 (0.54–2.33)	0.77
**Soil type**		
Silt	*Reference*	
Sand	2.61 (0.08–86.86)	0.59
Clay	1.25 (0.41–3.84)	0.69
**Current land use**		
Residential area	*Reference*	
Rice/agriculture field	5.11 (0.32–81.69)	0.25
Pasture land	19.46 (0.46–816.13)	0.12
Disused land	13.42 (0.59–307.30)	0.10

1 No B. pseudomallei positive isolates in “Temperate winter dry” climate zone

**Table 3:** Association of soil depth and isolation of *B*. *pseudomallei* by season.

	Proportion of *B*. *pseudomallei* positive samples (%)	Rate Ratio (95% CI)	p-value
**Cool dry season**			
30 cm	4/ 465 (0.9%)	1 (*Reference*)	
60 cm	10/ 465 (2.3%)	2.51 (0.64–9.83)	0.19
90 cm	12/ 465 (2.6%)	3.30 (0.86–12.65)	0.08
**Hot dry season**			
30 cm	5/321 (1.6%)	1 (*Reference*)	
60 cm	5/321 (1.6%)	1.00 (0.16–6.33)	1.00
90 cm	4/321 (1.3%)	0.77 (0.11–5.22)	0.79
**Monsoon season**			
30 cm	19/375 (5.1%)	1 (*Reference*)	
60 cm	13/375 (3.5%)	0.66 (0.25–1.76)	0.41
90 cm	14/375 (3.7%)	0.67 (0.26–1.75)	0.43

We identified *B*. *pseudomallei* in 67 (4.8%) samples collected in the tropical monsoon zone and 36 (2.1%) samples in the tropical winter dry zone (Fig 2 and S2 Table). We did not identify *B*. *pseudomallei* in any samples collected from the temperate dry winter zone. The tropical monsoon zone had a higher proportion of *B*. *pseudomallei*-positive samples [RR-2.26; 95% CI (0.21–6.21), p = 0.11] than the tropical winter dry zone but this was not statistically significant.

*B*. *pseudomallei* was isolated from 4.6% (57/1250) of samples collected in the monsoon season compared to 2.0% (31/1550) in the cool dry season and 1.4% (15/1070) in the hot dry season (Fig 2 and S2 Table). Soil samples positive for *B*. *pseudomallei* were more common when collected during the monsoon season [RR-2.28; 95% CI (0.70–7.38), p = 0.17] and, less common, in the hot dry season compared to those collected in the cool dry season [RR-0.70, 95% CI (0.19–2.56), p = 0.59] although these associations were not statistically significant (Table 2).

The most common soil type was silt [2590 samples (66.9%)], followed by clay [1200 samples (31.0%)] and sand [80 samples (2.1%)]. The proportion of samples positive for *B*. *pseudomallei* was higher among sandy soil [5/80 samples (6.3%)] compared to clay type (36/1200 samples [3.0%]) and silt type [62/2590 samples (2.4%)]. Soil samples positive for *B*. *pseudomallei* were more common in sandy soil [RR- 2.61, 95% CI (0.08–86.86), p = 0.59] and in clay type of soil [RR-1.25, 95% CI (0.41–3.84), p = 0.69] compared to the silt type but these associations were not statistically significant.

Of 3870 samples, 3150 (81.4%) were collected from agricultural land, 360 (9.3%) from disused land, 230 (5.8%) from residential areas and 130 (3.4%) from pasture land. Most *B*. *pseudomallei* positive cultures came from samples collected from agricultural land (70 samples (2.2%)), but the proportion of *B*. *pseudomallei*-positive samples was higher from pasture land (11 samples (8.5%)) and disused land (21 samples (5.8%)), whereas the proportion of positive samples from residential land was lower (1 sample (0.4%)). *B*. *pseudomallei* was isolated more often in soil samples collected from pasture land [RR-19.46; 95% CI (0.46–816.13), p = 0.12], disused land [RR-13.42; 95% CI (0.59–307.30), p = 0.10] and rice and agriculture land [RR-5.11; 95% CI (0.32–81.69), p = 0.25] compared to those collected from residential areas but these associations were not statistically significant. The *B*. *pseudomallei* isolation rate was 2.4% (28/1161) for both 30 and 60 cm and 2.6% (30/1161) for 90 cm depths (Fig 2). However, the effect of depth on isolation of *B*. *pseudomallei* varied between seasons (Table 3). In the cool dry season *B*. *pseudomallei* was isolated more often in soil samples collected at 60 cm [RR-2.51; 95% CI (0.64–9.83), p = 0.19] and 90 cm [RR-3.30; 95% CI (0.86–12.65), p = 0.08] compared to those collected at 30 cm but the associations were not statistically significant. In contrast, in the monsoon season *B*. *pseudomallei* was isolated less often in soil samples collected at 60 cm [RR-0.66; 95% CI (0.25–1.76), p = 0.41] and 90 cm [RR-0.67; 95% CI (0.26–1.75), p = 0.42] compared to those collected at 30 cm although the associations were not statistically significant. Pooled samples had a significantly higher overall positivity rate than individual samples (p = 0.02). Of the 387 pooled samples, 17 were positive. Thus of 31 sites from which *B*. *pseudomallei* was isolated, only half of the pooled samples 54.8% (17/31) had a positive culture. In one site, however, *B*. *pseudomallei* was isolated only in the pooled sample but not in any of the individual samples. We also identified *B*. *thailandensis*, a close relative of *B*. *pseudomallei*, in 72 of 387 (19%) sites and 135 out of 3870 (4%) samples in 13 states and regions (S3 Table). Both *B*. *pseudomallei* and *B*. *thailandensis* were identified in 4 of the 387 sites.

## Discussion

This study, the first of its kind in terms of coverage and sample size in Myanmar, revealed a widespread distribution of *B*. *pseudomallei* in soil. Our main interest was whether *B*. *pseudomallei* was present in the soil near human settlements. Therefore, we only collected soil samples from anthropogenically modified areas close to human settlements and native pristine soils were not included. We isolated *B*. *pseudomallei* in 103 samples from 31 locations across seven states and regions. All positive samples were located in the more densely populated central and southern coastal areas of the country. We did not isolate *B*. *pseudomallei* in samples from the northern and southernmost parts of the country. This was unexpected, as these locations have been predicted to be environmentally suitable for *B*. *pseudomallei* [1] and we are aware of a confirmed case of melioidosis most likely acquired in Kachin in the north of Myanmar (Tun Tun Win pers. comm. 2018). In addition, although we did not isolate *B*. *pseudomallei* in the southernmost province, Tanintharyi, which is located in the coastal area between the Andaman Sea and Thailand, a number of culture-confirmed human and animal cases of melioidosis have been reported in neighbouring provinces in western Thailand in recent years.[38] Possible explanations include the relative insensitivity of culture for detecting environmental *B*. *pseudomallei*, the uneven distribution of the organism in the environment, [39,40] and the relatively low number of samples tested in the sparsely populated South. It is thus quite possible that *B*. *pseudomallei* is also present in the northern and southern parts of Myanmar, but that we simply did not detect it in our study.

Most *B*. *pseudomallei* were isolated in the coastal regions of Myanmar, which corresponds to the tropical monsoon climate zone having a high annual rainfall between 2500 and 5000 mm (S4 Table). In the central plain region, which corresponds to the tropical dry winter zone with an annual rainfall between 500 and 1000 mm of annual rainfall, [41] isolation of *B*. *pseudomallei* was less common. In the temperate dry winter zone, in the northern part of the country, we did not isolate any *B*. *pseudomallei*. Rivers have been suggested as potential carriers and sentinels for *B*. *pseudomallei* [42] and the higher positivity rate for *B*. *pseudomallei* in the river delta of Ayeyawady region might be related to erosion of the soil, which harbour the organisms, during heavy rainfall, which is then transported by the main rivers and subsequently deposited downstream and in the coastal regions.

Although we isolated *B*. *pseudomallei* in all three seasons, a higher isolation rate was found in the rainy monsoon season. The monsoon season is also the time that most patients with melioidosis are diagnosed in other countries in the region including India, Nepal, Bangladesh, Thailand, Laos and Cambodia.[43–46]

*B*. *pseudomallei* was isolated in all three types of soils; sand, silt and clay. Although isolation was more frequent in sandy and clay type of soil compared to silt, the association was not statistically significant in our study. Previous studies have also reported a positive association of *B*. *pseudomallei* with sandy soil compared to clay.[18,47,48]

We found a higher proportion of *B*. *pseudomallei*-positive soil samples collected from agricultural land, pasture land and disused land compared with those collected from residential areas. The presence of *B*. *pseudomallei* in agricultural land including rice fields has frequently been described [17,19,49] and this is where most contact between soil and people is expected. Rice farmers are known to be at high risk for melioidosis.[20] In Myanmar, farmers use cattle and buffalo as draft animals throughout the country and these animals are typically grazed on the pasture land nearby agriculture land. The occurrence of *B*. *pseudomallei* in land disturbed by livestock animals has also been reported in an earlier study carried out in northern Australia.[50]

Samples collected at three different depths (30 cm, 60 cm and 90 cm) yielded a similar overall prevalence of *B*. *pseudomallei* in this study. The isolation of *B*. *pseudomallei* at the three soil depths varied between the seasons. We isolated *B*. *pseudomallei* more often at deeper depths (60 cm, 90 cm) during the cool dry season but more often at the shallower depth (30 cm) during the monsoon season (Table 3). This is in line with an earlier study in Australia.[51] but differs from a study carried out in Laos where higher isolation rates were found at soil depths greater than 30 cm.[49] However, based on these results we believe that it remains good practice to collect samples at all 3 different depths. It might be advisable to collect samples from depths beyond 30 cm in the hot dry and cool dry seasons but this may vary from place to place and needs further confirmation.

As part of this study, we explored the potential of pooling multiple soil samples to simplify the procedures for isolation of *B*. *pseudomallei*, but this method was only partially successful. Pooled samples had a significantly higher overall positivity rate than individual samples, but failed to isolate *B*. *pseudomallei* from 14 of 31 sites where *B*. *pseudomallei* was isolated from one or more individual samples. On the other hand, there was one site where the pooled sample was positive for *B*. *pseudomallei* whereas the nine individual samples were all negative. This also highlights the difficulty of isolating *B*. *pseudomallei* from environmental samples and the advisability of using multiple samples and methods before regarding any site as negative.[40] We did not identify both *B*. *pseudomallei* and *B*. *thailandensis* in the same sample in our study, suggesting there might have been competition between the species as suggested by a previous study.[52] However, other studies have found the two species present in the same samples [53] and, since the ability to detect this is likely to be highly method dependent and our study was designed primarily to detect the presence of *B*. *pseudomallei*, it would be inappropriate to draw and conclusions on this point. Soil pH was not tested in our study, and therefore we could not investigate the effect of soil pH on the occurrence of *B*. *pseudomallei* in the soil samples. However, recent data from an agricultural company (S1 Fig) indicates that the soil pH in the southern part of the country, where most *B*. *pseudomallei* was identified, was generally within the range of 3.7 to 4.8, while soil in the central part of Myanmar, where only a few samples with *B*. *pseudomallei* were identified, has a higher pH, above 5.4.

### Advantage and limitations

The failure to isolate *B*. *pseudomallei* in certain areas does not mean that it was not present and the results of this study probably present an underestimate of the true distribution of *B*. *pseudomallei* across Myanmar for several reasons. First, we only collected samples from 9 points in each site compared to 100 points per site as suggested in the consensus guideline.[30] With limited resources available, we chose to select more geographically dispersed sites with fewer samples in order to obtain a better overview of the geographical distribution nationally. Secondly, we omitted a number of areas because of limited access or armed conflict, potentially missing positive locations. Third, as samples were only collected at one time point for each province, each province was only sampled in a single season, which may have reduced our ability to detect *B*. *pseudomallei*. Fourth, the culture method used in the study, although based on a consensus of international experts on melioidosis, is known to have a lower sensitivity for the detection of *B*. *pseudomallei* in soil when compared with molecular methods such as PCR.[40] And finally, the fact that pooled samples often tested negative while one or more of the individual samples of the same sites tested positives, and a pooled sample tested positive while all individual samples tested negative, shows clearly the ‘hit and miss’ sensitivity of the culture method. Negative findings are therefore never an assurance that *B*. *pseudomallei* is not present in the area.

The widespread distribution of *B*. *pseudomallei* is a worrying finding given the rarity with which melioidosis is diagnosed in the country, and suggests that many cases of melioidosis are going undiagnosed. This is particularly likely amongst agricultural workers, who comprise 65% percent of the population in Myanmar with rice being the main cultivated crop.[54,55] In addition, diabetes mellitus is the major predisposing factor for melioidosis and the prevalence of diabetes is rapidly increasing in Myanmar.[56] The incidence of melioidosis is associated with heavy rainfall in the monsoon season [45,46] and people engaging in agricultural work during the monsoon season are at an increased risk. Ayeyawady province has the highest proportion of paddy fields and the highest proportion of *B*. *pseudomallei*-positive sites. In this regard it is worthy of note that a recent serological study carried out in 124 febrile patients in the delta area of Ayeyawady province observed a 3.2% positivity rate consistent with possible *B*. *pseudomallei* infection.[57] In addition to its widespread distribution, a recent whole genome sequencing (WGS) study demonstrated the genetic diversity of *B*. *pseudomallei* isolates from Myanmar suggesting long-term endemicity of *B*. *pseudomallei* in the country.[36]

The fact that *B*. *pseudomallei* is widely distributed in the soil in Myanmar is not surprising. The environmental suitability for the organism is high in large areas of south and south-east Asia, and the predicted burden of melioidosis in 2015 was high for the entire region; India (52,506 cases/31,425 deaths), Bangladesh (16,931/9,454), Laos (420/260), Thailand (7,572/2,838): the corresponding figures for Myanmar were 6,247 cases and 3,687 deaths.[1]

How can these patients be identified? It is difficult to diagnose melioidosis clinically as it has no pathognomonic clinical signs and symptoms. It has been nicknamed the ‘great imitator’ because of its variable presentation and the wide differential diagnosis. Laboratory confirmation is therefore essential, but in most rural areas of Myanmar where melioidosis is expected to be most common, microbiology laboratories are not available. And even in laboratories where cultures are performed, *B*. *pseudomallei* is often misidentified as a contaminant or other species, especially by laboratory staff who are not familiar with the organism and where selective media and specific identification tests are not in-use.[12,58]

In Thailand and Laos, two neighbouring countries, melioidosis was only recognized as a major cause of community acquired septicaemia after researchers actively looked for the disease by establishing laboratory facilities to culture and identify *B*. *pseudomallei*.[24,38,59,60] In northeast Thailand melioidosis is now the second most common cause of community-acquired bacteraemia (19.3%) after *E*. *coli* (23.1%), and the number of deaths caused by melioidosis is comparable to deaths related to tuberculosis and higher than that caused by other common infectious diseases such as malaria, dengue, measles and leptospirosis. [61] The number of deaths from melioidosis in Thailand has, however, been significantly under-estimated by the National Notifiable Diseases Surveillance System until recently.[62] In Laos, the number of melioidosis cases detected annually also increased dramatically after 1999 when researchers began specifically looking for it.[59] It is very likely that the situation in Myanmar is similar and that the number of patients diagnosed with melioidosis will increase substantially when clinicians start actively looking for the disease, diagnostic microbiology services are strengthened, and laboratory staff gain experience in the detection and identification of *B*. *pseudomallei*. Recent training initiatives in Cambodia and Laos have been undertaken in order to raise awareness of this disease and similar events should be considered in Myanmar.[63,64]

## Conclusion

Melioidosis is a neglected disease in many tropical and sub-tropical regions. This is the first environmental survey for *B*. *pseudomallei* conducted in all states and regions of Myanmar. The findings confirm that *B*. *pseudomallei* is widely distributed in central parts of Myanmar. Due to limited diagnostic facilities, and the lack of awareness among many clinical staff, particularly in rural areas of Myanmar where the disease is thought to be most common, it is very likely that melioidosis is substantially under-diagnosed and under-reported in Myanmar.

These findings should alert clinicians in Myanmar to consider melioidosis as one of the potential causes of fever, sepsis, pneumonia and abscesses, in particular during the rainy season in patients in regular contact with soil and those with underlying risk factors like diabetes. Clinical awareness should be accompanied with the provision of diagnostic facilities. In settings without microbiology facilities, a rapid diagnostic test for melioidosis has the potential to improve the accuracy and timeliness of diagnosis and treatment of melioidosis and thus save lives.[65,66]

In addition, further clinical studies to explore the epidemiology of melioidosis in Myanmar, prioritising regions where *B*. *pseudomallei* has been found in soil, should be initiated.

## Supporting information

S1 Table *Burkholderia pseudomallei* positive and negative townships in 15 states and regions of Myanmar. (DOCX)

S2 Table Details of culture sample result and seasons, soil depths, soil types, current land use and climate zones. (DOCX)

S3 Table Positivity of *Burkholderia thailandensis* in 15 states and regions of Myanmar. (DOCX)

S4 Table Positivity of *Burkholderia pseudomallei* in 15 states and regions with annual rainfall data. (DOCX)

S1 Fig Soil pH map of Myanmar (Source: Agrocares, www.agrocares.com). (TIF)
