# Supplementary material for: Geographical distribution of Burkholderia pseudomallei in soil in Myanmar
Source: PLoS Negl Trop Dis. 2021 May 24;15(5):e0009372. doi: 10.1371/journal.pntd.0009372 (PMC8143414; doi:10.1371/journal.pntd.0009372)
Supplement: S1 Table — (DOCX) [file pntd.0009372.s001.docx]

**S1 Table. *Burkholderia pseudomallei* positive and negative townships in 15 states and regions of Myanmar**

| States and regions | *B. pseudomallei* positive townships, n=21 | *B. pseudomallei* negative  townships, n=140 |
| --- | --- | --- |
| Ayeyawady | Bogale, Hinthada, Kangyidaunt, Maubin, Pyapon, Thabaung | Danubyu, Ingapu, Kyaunggon, Labutta, Myanaung, Myaungmya, Ngapudaw, Pantanaw, Yegyi |
| Kayin | Hpa-An, Kawkareik, Myawaddy | Kyainseikgyi, Thandaunggyi |
| Bago | Paukkhaung, Paungde, Shwedaung, Zigon | Daik-U, Kawa, Kyaukkyi, Letpadan, Nyaunglebin, Okpho, Penwegon, Phyu, Swar, Taungoo, Thanatpin, Waw, Yedashe |
| Rakhine | Gwa, Kyaukphyu | Ramree, Thandwe, Toungup |
| Mon | Kyaikmaraw, Thanbyuzayat | Bilin, Chaungzon, Kyaikto, Mawlamyine, Paung, Thaton, Ye |
| Yangon | Hmawbi, Kyauktan, Twantay | Dala, Hlaingtharya, Hlegu, Htantabin, Kawhmu, Kayan, Kungyangon, Shwepyithar, Taikkyi, Thanlyin, Thongwa |
| Magway | Magway | Aunglan, Chauk, Myaing, Myothit, Natmauk, Pakokku, Pauk, Pwintbyu, Salin, Sinbaungwe, Taungdwingyi, Yenangyaung, Yesagyo |
| Kachin |  | Bhamo, Hpakant, Kamine, Machanbaw, Mogaung, Mohnyin, Momauk, Myitkyina, Nanmati, Putao, Waingmaw |
| Sagaing |  | Ayadaw, Chaung-U, Depayin, Kale, Kalewa, Kawlin, Khin-U, Mawlaik, Monywa, Myinmu, Pale, Sagaing, Salingyi, Shwebo, Taze, Wetlet, Wuntho, Ye-U, Yinmabin |
| Mandalay |  | Kyaukse, Madaya, Mahlaing, Meiktila, Myingyan, Natogyi, Pyawbwe, Pyinoolwin, Singu, Taungtha, Thazi, Wundwin |
| Kayar |  | Bawlakhe, Demoso, Hpasawng, Loikaw |
| Tanintharyi |  | Bokpyin, Dawei, Kawthoung, Launglon, Myeik, Palaw, Tanintharyi, Thayetchaung, Yebyu |
| Shan |  | Hopong, Hsihseng, Hsipaw, Kalaw, Kyaukme, Lashio, Loilem, Nansang, Naungkhio, Nyaungshwe, Phekon, Pindaya, Pinlaung, Pinlon, Shwenyaung, Taunggyi, Yatsuk |
| Naypyitaw |  | Lewe, Poke Ba Thi Ri, Pyinmana, Tatkon, Zay Yar Thi Ri |
| Chin |  | Falam, Hakha, Paletwa, Tedim, Thantlang |
