# Supplementary material for: Geographical distribution of Burkholderia pseudomallei in soil in Myanmar
Source: PLoS Negl Trop Dis. 2021 May 24;15(5):e0009372. doi: 10.1371/journal.pntd.0009372 (PMC8143414; doi:10.1371/journal.pntd.0009372)
Supplement: S2 Table — (DOCX) [file pntd.0009372.s002.docx]

**S2 Table. Details of culture sample result and seasons, soil depths, soil types, current land use and climate zones**

|  | **Total**  N=3870 | ***B. pseudomallei***  n=103 | ***B. thailandensis***  n=135 | **Other flora**  n=3068 | **No growth**  n=564 |
| --- | --- | --- | --- | --- | --- |
| **Climate zones** |  |  |  |  |  |
| Tropical dry winter /dry steppe | 1700 (43.9%) | 36/1700 (2.1%) | 48/1700 (2.8%) | 1364/1700 (80.2%) | 252/1700 (14.8%) |
| Tropical monsoon | 1400 (36.2%) | 67/1400 (4.8%) | 76/1400 (5.4%) | 986/1400 (70.4%) | 271/1400 (19.4%) |
| Temperate dry winter | 770 (19.9%) | 0/770 | 11/770 (1.4%) | 718/770 (93.3%) | 41/770 (5.3%) |
|  |  |  |  |  |  |
| **Season** |  |  |  |  |  |
| Cool, dry | 1550 (40.1%) | 31/1550 (2.0%) | 70/1550 (4.5%) | 1245/1550 (80.3%) | 204/1550 (1.32%) |
| Hot, dry | 1070 (27.6%) | 15/1070 (1.4%) | 17/1070 (1.6%) | 971/1070 (90.7%) | 67/1070 (6.3%) |
| Monsoon | 1250 (32.3%) | 57/1250 (4.6%) | 48/1250 (3.8%) | 852/1250 (68.2%) | 293/1250 (23.4%) |
|  |  |  |  |  |  |
| **Soil depth** |  |  |  |  |  |
| 30 cm | 1161 (30.0%) | 28/1161 (2.4%) | 41/1161 (3.5%) | 941/1161 (81.1%) | 151/1161 (13%) |
| 60 cm | 1161 (30.0%) | 28/1161 (2.4%) | 36/1161 (3.1%) | 928/1161 (79.9%) | 169/1161 (14.6%) |
| 90 cm | 1161 (30.0%) | 30/1161 (2.6%) | 43/1161 (3.7%) | 882/1161 (76.0%) | 206/1161 (17.7%) |
| pooled sample | 387 (10.0%) | 17/387 (4.4%) | 15/387 (3.9%) | 317/387 (81.9%) | 38/387 (9.8%) |
|  |  |  |  |  |  |
| **Soil type** |  |  |  |  |  |
| Silt | 2590 (66.9%) | 62/2590 (2.4%) | 93/2590 (3.6%) | 2052/2590 (79.2%) | 383/2590 (14.0%) |
| Sand | 80 (2.1%) | 5/80 (6.3%) | 2/80 (2.5%) | 65/80 (81.3%) | 8/80 (10.0%) |
| Clay | 1200 (31.0%) | 36/1200 (3.0%) | 40/1200 (3.3%) | 951/1200 (79.3%) | 173/1200 (14.4%) |
|  |  |  |  |  |  |
| **Current land use** |  |  |  |  |  |
| Residential area | 230 (5.9%) | 1/230 (0.4%) | 7/230 (3.0%) | 166/230 (72.2%) | 56/230 (24.4%) |
| Rice/ Agriculture | 3150 (81.4%) | 70/3150 (2.2%) | 112/3150 (3.6%) | 2545/3150 (80.8%) | 423/3150 (13.4%) |
| Pasture land | 130 (3.4%) | 11/130 (8.5%) | 2/130 (1.5%) | 80/130 (61.5%) | 37/130 (28.5%) |
| Disused land | 360 (9.3%) | 21/360 (5.8%) | 14/360 (3.9%) | 277/360 (76.9%) | 48/360 (13.3%) |
|  |  |  |  |  |  |
