# Supplementary material for: Geographical distribution of Burkholderia pseudomallei in soil in Myanmar
Source: PLoS Negl Trop Dis. 2021 May 24;15(5):e0009372. doi: 10.1371/journal.pntd.0009372 (PMC8143414; doi:10.1371/journal.pntd.0009372)
Supplement: S3 Table — (DOCX) [file pntd.0009372.s003.docx]

**S3 Table. Positivity of *Burkholderia thailandensis* in 15 states and regions of Myanmar**

| **State and regions** |  | **Site Positivity** |  | **Sample Positivity** |
| --- | --- | --- | --- | --- |
| Ayeyawady |  | 10/36 (28) |  | 20/360 (6) |
| Kayin |  | 7/24 (29) |  | 11/240 (5) |
| Bago |  | 6/37 (16) |  | 10/370 (3) |
| Rakhine |  | 2/13 (15) |  | 8/130 (6) |
| Mon |  | 13/26 (50) |  | 29/260 (11) |
| Yangon |  | 7/36 (19) |  | 10/360 (3) |
| Magway |  | 4/29 (14) |  | 6/290 (2) |
| Kachin |  | 8/28 (29) |  | 11/280 (4) |
| Sagaing |  | 5/42 (12) |  | 5/420 (1) |
| Mandalay |  | 4/24 (17) |  | 10/240 (4) |
| Kayar |  | 2/9 (22) |  | 2/90 (2) |
| Tanintharyi |  | 2/20 (10) |  | 11/200 (6) |
| Shan |  | 2/36 (6) |  | 2/360 (1) |
| Nay Pyi Taw |  | 0/9 |  | 0/90 |
| Chin |  | 0/18 |  | 0/180 |
| **Total** |  | 72/387 (19) |  | 135/3870 (3) |

*Data are shown in frequency (percentage)*
