# Supplementary material for: Geographical distribution of Burkholderia pseudomallei in soil in Myanmar
Source: PLoS Negl Trop Dis. 2021 May 24;15(5):e0009372. doi: 10.1371/journal.pntd.0009372 (PMC8143414; doi:10.1371/journal.pntd.0009372)
Supplement: S4 Table — (DOCX) [file pntd.0009372.s004.docx]

**S4 Table. Positivity of *Burkholderia pseudomallei* in 15 states and regions with annual rainfall data**

| **States and regions** | **Average annual rainfall (mm)*** | **Site positivity** | **Sample positivity** |
| --- | --- | --- | --- |
| Ayeyawady | 2888 | 9/ 36 (25.0%) | 34 /360 (9.4%) |
| Kayin | 684 | 5/ 24 (20.8%) | 11/ 240 (4.6%) |
| Bago | 1598 | 6/ 37 (16.2%) | 18/ 370 (4.9%) |
| Rakhine | 5064 | 2/ 13 (15.4%) | 15/ 130 (11.5%) |
| Mon | 5315 | 3/ 26 (11.5%) | 7/ 260 (2.7%) |
| Yangon | 2681 | 4/ 36 (11.1%) | 13/ 360 (3.6%) |
| Magway | 844 | 2/ 29 (6.9%) | 5/ 290 (1.7%) |
| Kachin | 3252 | 0/ 28 | 0/ 280 |
| Sagaing | 1820 | 0/ 42 | 0/ 420 |
| Mandalay | 826 | 0/ 24 | 0/ 240 |
| Kayar | 1162 | 0/ 9 | 0/ 90 |
| Tanintharyi | 5440 | 0/ 20 | 0/ 200 |
| Shan | 1321 | 0/ 36 | 0/ 360 |
| Naypyitaw | 1189 | 0/ 9 | 0/ 90 |
| Chin | 1471 | 0/ 18 | 0/ 180 |
| **Grand total** | 35554 | 31/ 387 (8.0%) | 103/3870 (2.7%) |

**Based on historic data from 1980 - 2010*.
