# Supplementary figures and images for: Geographical distribution of Burkholderia pseudomallei in soil in Myanmar
Source: PLoS Negl Trop Dis. 2021 May 24;15(5):e0009372. doi: 10.1371/journal.pntd.0009372 (PMC8143414; doi:10.1371/journal.pntd.0009372)

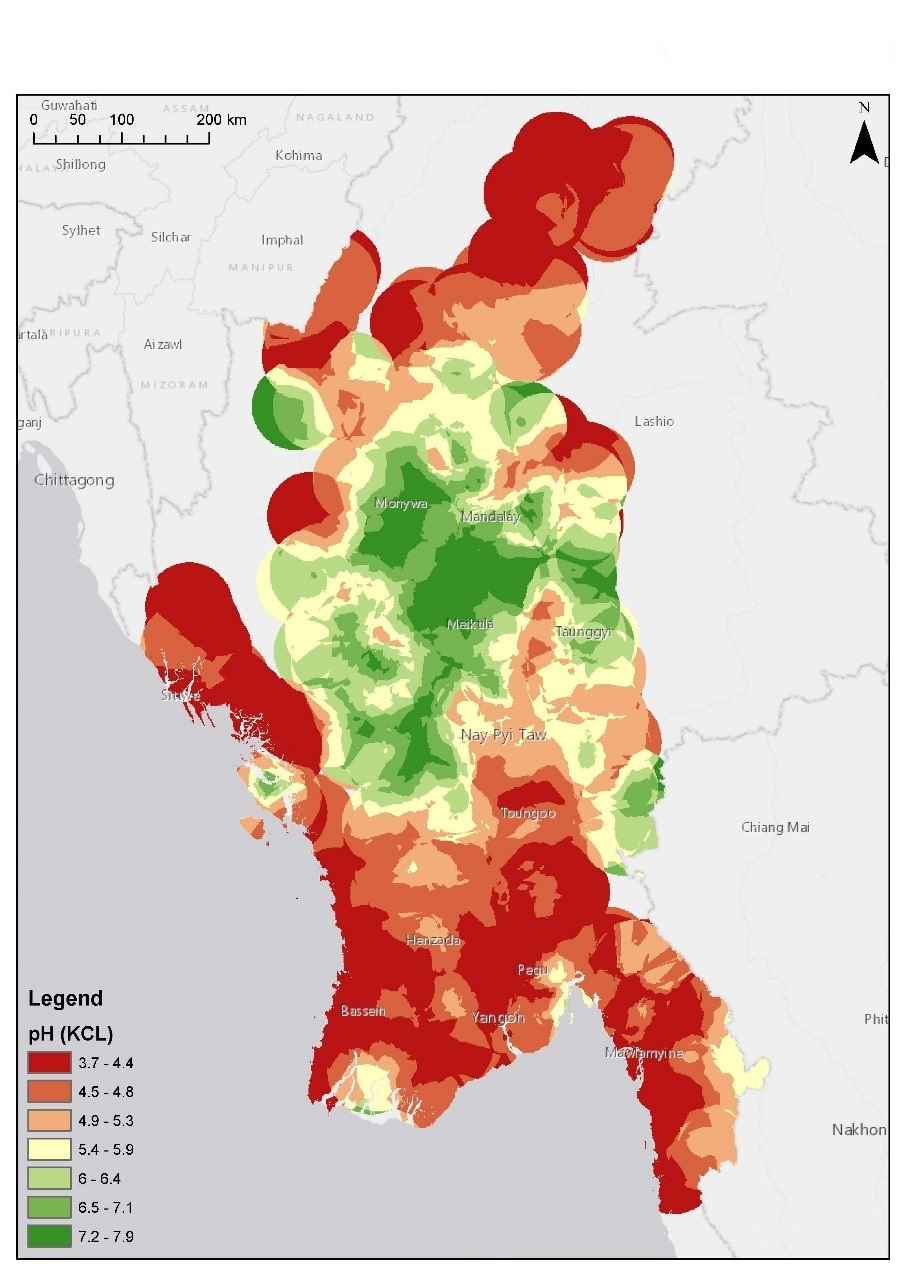

Supplement: S1 Fig — (TIF) [file pntd.0009372.s005.tif]
